# Supplementary material for: Observation of current-induced switching in non-collinear antiferromagnetic IrMn3 by differential voltage measurements
Source: Nat Commun. 2021 Jun 22;12:3828. doi: 10.1038/s41467-021-24237-y (PMC8219769; doi:10.1038/s41467-021-24237-y)
Supplement: Supplementary file 1 — Supplementary Information [file 41467_2021_24237_MOESM1_ESM.pdf]

Supplementary Information

Observation of current-induced switching in non-collinear antiferromagnetic $IrMn_3$ by differential voltage measurements

Sevdenur Arpaci [1,2†], Victor Lopez-Dominguez [1†*], Jiacheng Shi [1], Luis Sánchez-Tejerina [3], Francesca Garesci [4], Chulin Wang [1], Xueting Yan [1], Vinod K. Sangwan [5], Matthew Grayson [1,2], Mark C. Hersam [1,2,5,6], Giovanni Finocchio [3*], Pedram Khalili Amiri [1,2*]

[1] Department of Electrical and Computer Engineering, Northwestern University, Evanston, Illinois 60208, United States of America

[2] Graduate Program in Applied Physics, Northwestern University, Evanston, Illinois 60208, United States of America

[3] Department of Mathematical and Computer Sciences, Physical Sciences and Earth Sciences, University of Messina, Messina 98166, Italy

[4] Department of Engineering, University of Messina, Messina 98166, Italy

[5] Department of Materials Science and Engineering, Northwestern University, Evanston, Illinois 60208, United States of America

[6] Department of Chemistry, Northwestern University, Evanston, Illinois 60208, United States of America

[†] These authors contributed equally to this work.

[*] Correspondence and requests for materials should be addressed to V.L.-D., G.F., or P.K.A.

[*] Email: victor@northwestern.edu , gfinocchio@unime.it , pedram@northwestern.edu

Supplementary Note 1: Structural characterization

To characterize the composition and crystallographic structure of the co-sputtered $IrMn_3$ films, we performed x-ray diffraction (XRD) measurements (see Supplementary Fig. 1) on $Si/SiO_2/Pt(5)/IrMn_3(10)/MgO$ multilayer structures similar to the ones used in the experiments. The results show a central peak consistent with an $IrMn_3$ phase with a (111) texture. Peaks associated with Pt, in particular the diffraction peak expected from the (111) plane at 40°, are overlapped by the broad $IrMn_3$ detected peak. This feature and the absence of additional diffraction peaks for Pt suggest that Pt is also (111) texturized. In addition, the absence of the (001) diffraction peak suggests a γ-$IrMn_3$ polycrystalline phase for the $IrMn_3$ micropillar [1, 2].

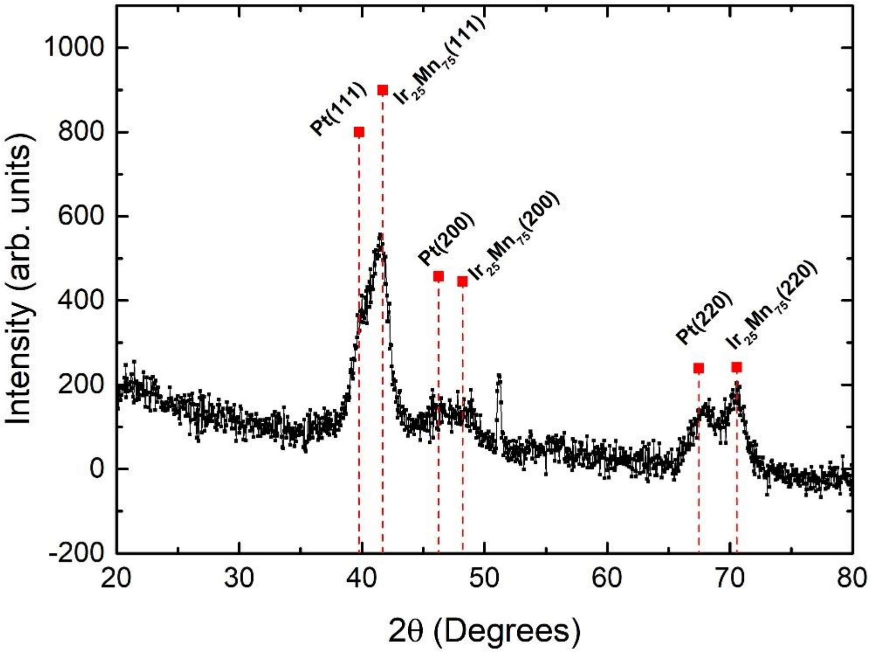

Supplementary Fig. 1. XRD measurement of $Si/SiO_2/Pt(5)/IrMn_3(10)/MgO$, red dashed lines showing the position of the peaks for Pt and $IrMn_3$.

Supplementary Note 2: Magnetic characterization

In order to optimize the growth parameters and study the magnetic characteristics of the co-sputtered $IrMn_3$ films, we constructed a series of Ta(5)/Pt(8)/Co(0.8)/Pt(1)/$IrMn_3$(t)/MgO(2.5) (thickness is expressed in nanometers) multilayers. This structure is expected to provide exchange bias in the perpendicularly magnetized Co layer without any annealing process [3-6]. The role of the intermediate Pt layer is to increase the interfacial perpendicular anisotropy of Co in addition to avoiding the diffusion of Mn atoms into the Co layer. More importantly, this test structure was chosen since the $IrMn_3$ layer is interfaced with Pt and MgO, closely resembling the structure that was used in our transport measurements. The hysteresis loop measured for the case of 7 nm $IrMn_3$ thickness by vibrating sample magnetometry (VSM) is shown in Supplementary Fig. 2. The loop was measured by applying a magnetic field perpendicular to the sample plane between -500 and 500 Oe, and indicates an exchange bias field of ~170 Oe.

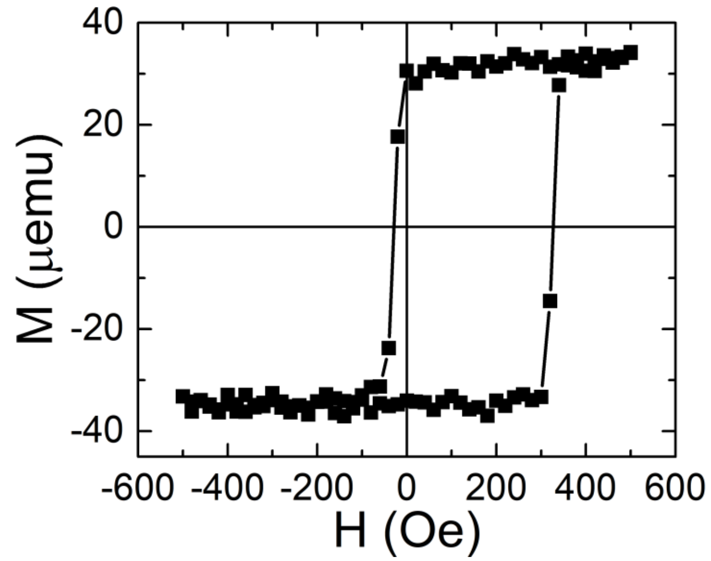

Supplementary Fig. 2. Out-of-plane VSM measurement of Ta(5)/Pt(8)/Co(0.8)/Pt(1)/$IrMn_3$(7)/MgO (thickness in nanometers) in the field range between -500 Oe to 500 Oe, indicating an exchange bias field of ~170 Oe.

Supplementary Note 3: Raw switching data for the device shown in the main text

In this Supplementary Note the raw data, without subtracting the background slope of the output voltage, of Figs. 2 and 3 of the main text are shown.

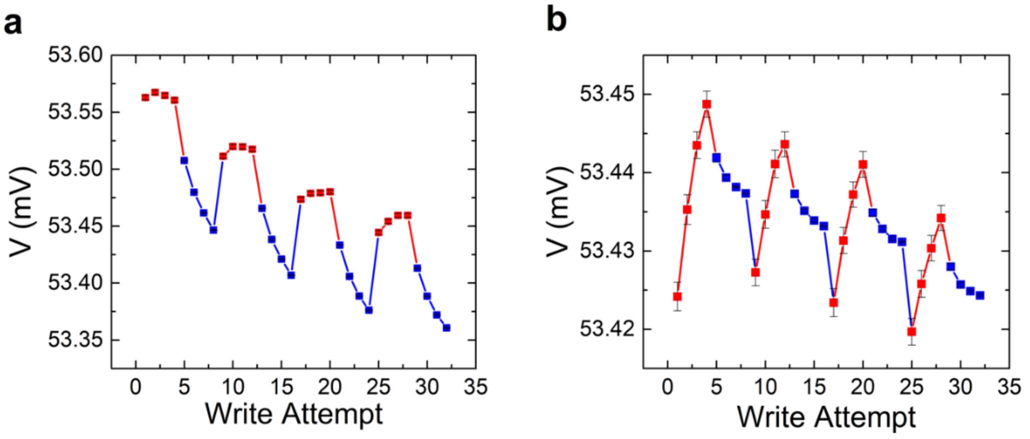

Supplementary Fig. 3. Measured $V^{2\text{-}3}_{Read}$ for the 90˚ degree switching scheme. a Measured voltage variation when a 20 mA writing pulse is applied in the 90˚ configuration. Red squares correspond to when the writing pulse is applied from electrode 1 to 4 and blue points from electrode 6 to 2 (the arm with the $IrMn_3$ pillar). **b** Measured voltage variation when the 20 mA writing pulse is applied from electrode 1 to 4 (red points) and electrode 5 to 3 (blue points). Each data point corresponds to the mean value of 170 consecutive measurements and the error bar to the standard deviation

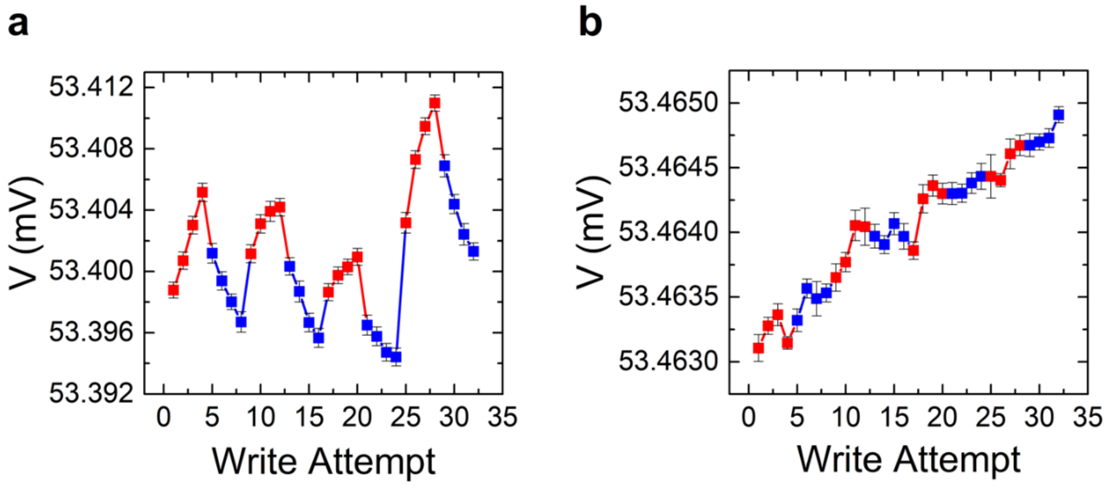

Supplementary Fig. 4. Measured $V^{2\text{-}3}_{Read}$ for the (vertical) 180˚ switching scheme. a Red squares correspond to when the writing pulse is applied from electrode 2 to 6 and blue points from electrode 6 to 2 (the arm with the $IrMn_3$ pillar). **b** Measured voltage variation when the 20 mA writing pulse is applied from electrode 3 to 5 (red points) and electrode 5 to 3 (blue points), corresponding to the Pt-only arm. Each data point corresponds to the mean value of 1 consecutive measurements and the error bar to the standard deviation

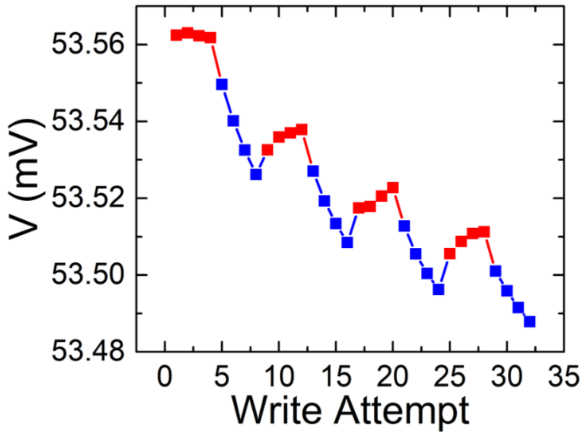

Supplementary Fig. 5. Measured $V^{2\text{-}3}_{Read}$ for the (horizontal) 180˚ switching scheme. Measured voltage variation when a 20 mA writing pulse is applied in the 180˚ configuration. Red squares correspond to when the writing pulse is applied from electrode 1 to 4 and blue points from electrode 4 to 1. Each data point corresponds to the mean value of 170 consecutive measurements and the error bar to the standard deviation

Supplementary Note 4: Results from 4 μm Device

In addition to the device of 6 μm diameter shown in the main text, we performed the same experiments in a device with a 4 μm diameter $IrMn_3$ pillar. In this case, the main switching experiments were performed using a slightly larger current amplitude, 22 mA, corresponding to a current density of 44 MA/cm^2. The results are shown in Supplementary Fig. 6, for the cases when the write current pulse is applied between the electrodes 2-6 (Supplementary Fig. 6a), 1-4 (Supplementary Fig. 6b), as well as 1-4 and 2-6 (90˚ switching shown in Supplementary Fig. 6c). For this current amplitude, we did not observe any switching behavior in the differential voltage when the write pulse was applied in the Pt-only arm, indicating that all switching signals in Supplementary Fig. 6 have a magnetic origin.

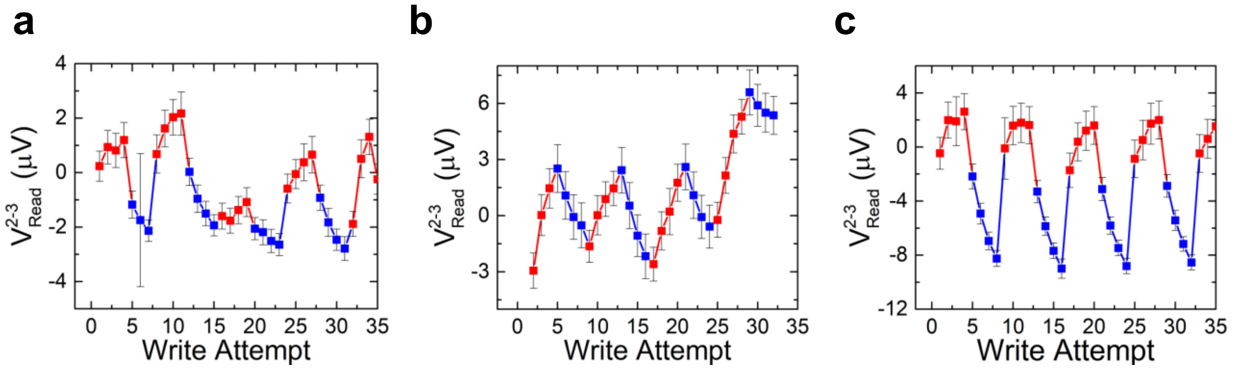

Supplementary Fig. 6. Switching behavior at 22 mA write current amplitude for a 4 μm diameter $IrMn_3$ micro-pillar device. **a** 180˚ switching experiment when the current pulse is applied between the electrodes 2-6 (red points, I^{2-6}_{write}), and the reverse current direction (blue points, I^{6-2}_{write}). **b** 180˚ switching configuration where the current pulse is applied in the longest arm of the device (I^{1-4}_{write} corresponding to red points, and I^{4-1}_{write} corresponding to blue points). **c** 90˚ switching experiment for a current pulse applied between electrodes 1 and 4 (red points, I^{1-4}_{write}), and between electrodes 6 and 2 (I^{6-2}_{write} , blue points). Each data point corresponds to the mean value of 170 consecutive measurements and the error bar to the standard deviation

The switching behavior is qualitatively similar to those obtained for the 6 μm diameter device in the main text, i.e. a sawtooth pattern indicating that the switching takes place by thermally assisted domain wall motion. A difference with the results shown in the main text for the 6 μm diameter device is the reduction of the differential voltage in the three studied configurations. This may be attributable to the reduction of AFM material size, implying a decrease of the magnetoresistance signal from the AFM. Nonetheless, similar to the 6 μm device shown in the main text, the 90˚ switching configuration provides the largest readout voltage in this case as well.

Supplementary Note 5: Pt-only double-cross results

To further verify the magnetic origin of the switching signal in the studied $IrMn_3$/Pt devices, we performed the same differential voltage measurements in double-cross devices only containing Pt, with no $IrMn_3$ pillar on either cross. When 20 mA write current pulses were applied to this device in both 90˚ and 180˚ configurations (same cases as the $IrMn_3$ micropillars shown in Figs. 2 and 3 of the main text), no switching behavior was observed from the differential voltage readings, as shown in Supplementary Fig. 4. This confirms that the switching measured in the $IrMn_3$/Pt devices has a magnetic origin, and is not related to any intrinsic mechanism in the Pt layer for current pulses with an amplitude of 20 mA or lower.

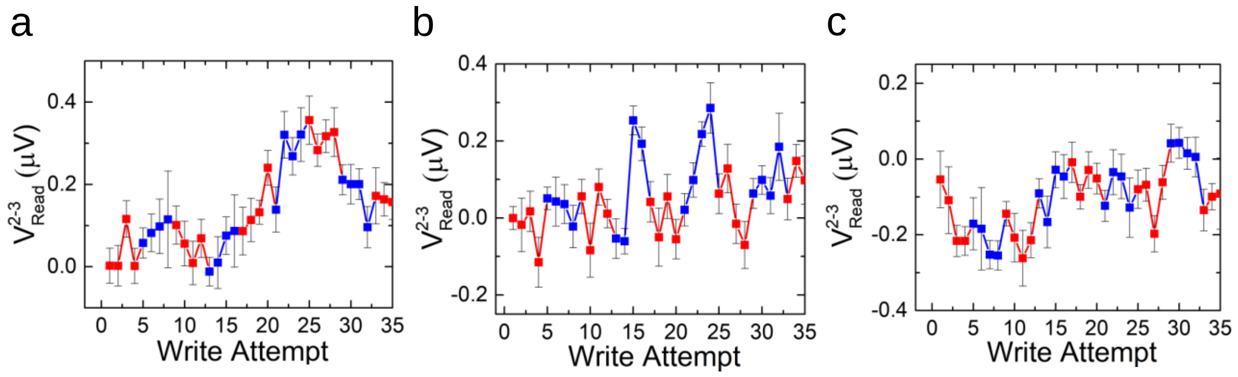

Supplementary Fig. 7. Control experiments on a Pt double-cross device (without $IrMn_3$ pillar) for the three studied switching schemes using 20 mA write pulses. **a** 90˚ switching scheme when the current pulse is applied between electrodes 1 and 4 (red points) or between electrodes 6 and 2 (blue points), **b** 180˚ switching scheme, applying the pulse between electrodes 2 and 6 (red points) and in the reverse direction (blue points) and, **c** 180˚ switching scheme, applying the write current between electrodes 1 and 4 (red points) and reversing the pulse direction (blue points). Each data point corresponds to the mean value of 170 consecutive measurements and the error bar to the standard deviation

Supplementary Note 6: Current-induced temperature increase in the Pt layer

To better understand the possible role of temperature increase during switching in our devices, we performed experiments to estimate the temperature increase induced by the write current pulse flowing in the Pt layer. For this purpose, we carried out experiments in a double-cross device without the $IrMn_3$ pillar, having the same Pt thickness as the devices shown in the main text.

First, we directly measured the temperature dependence of the device resistance, defined as, $\alpha = d(R/R_0)/dT$, where α is the thermal coefficient, T is the temperature, R_0 is the resistance at room temperature, and R is the (temperature-dependent) resistance of the device. Using this method we obtained an approximately linear resistance change with a thermal coefficient of $7.81 \times 10^{-4}\ K^{-1}$, as shown in Supplementary Fig. 8a. Next, the resistance change of the device was measured after applying writing currents with different amplitudes ranging from 1 to 10 mA. The results, shown in Supplementary Fig. 8b, indicate that the resistance increases approximately linearly with the square of the current amplitude. In this manner, using the calibrated thermal coefficient along with the resistance change measured at 10 mA amplitude, we estimated a device temperature increase of ~ 25 K. In the case of a 20 mA amplitude pulse (which is close to the write currents shown in the main manuscript) we can extrapolate to an estimated temperature increase in the film as high as ~ 96 K.

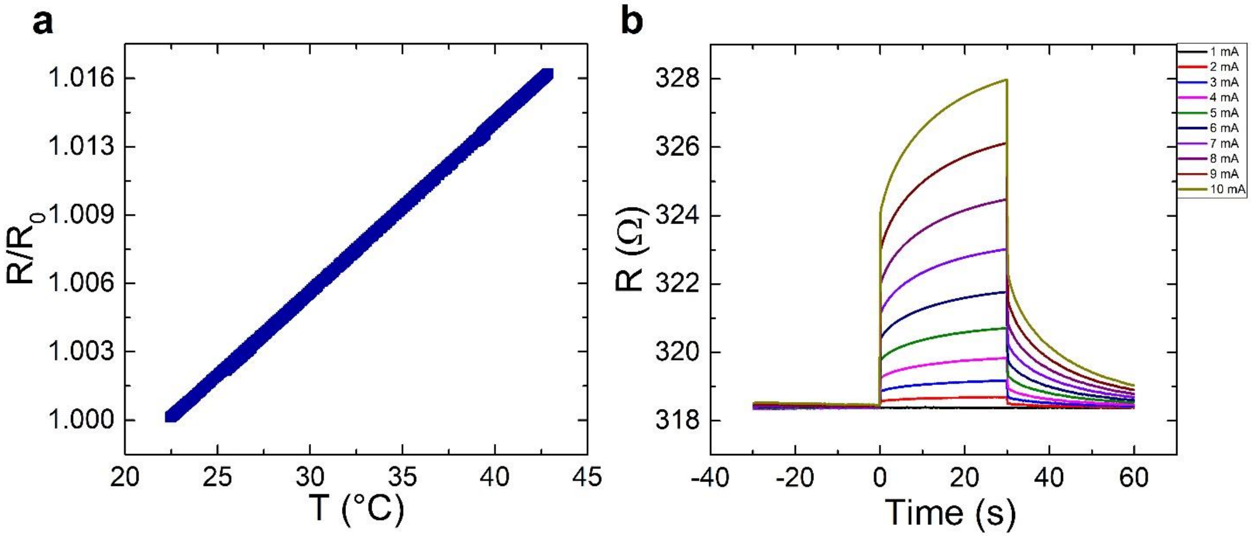

Supplementary Fig. 8. a Resistance change for a Pt double-cross device as a function of temperature. **b** Resistance change after the application of a writing current of 30 s width and different amplitudes.

Supplementary Note 7: Differential voltage measurement using an operational amplifier

We have implemented an alternative differential voltage measurement technique using a resistive voltage divider circuit combined with an operational amplifier (OA). The voltage divider circuit uses two resistors in addition to the double-cross device, as shown in Supplementary Fig. 9a. By choosing the resistance values such that the ratio between the external resistors and the resistances of both arms in our device (with and without the $IrMn_3$ pillar) are the same, the voltage inputs to the OA will be at the same potential ($V_+ = V_-$), similar to a Wheatstone bridge circuit in its balanced condition. Once write currents are applied using the current source, the resistance of the arm with the $IrMn_3$ pillar switches, resulting in resistance changes by a larger amount than the one in the arm without the $IrMn_3$ pillar. This throws the circuit off-balance ($V_+ \neq V_-$), generating a detectable voltage difference at the output of the OA. This method provides a larger output signal than those in the main manuscript, as a consequence of the OA voltage gain.

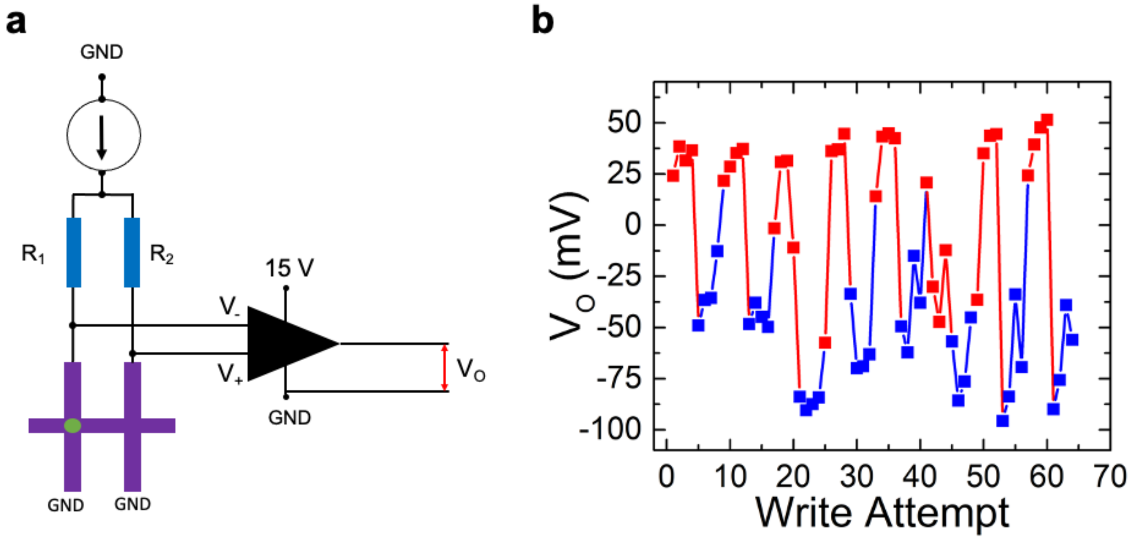

Supplementary Fig. 9. Differential voltage measurement using an operational amplifier on a double-cross device with a single $IrMn_3$ pillar. a Schematic of the measurement circuit with the resistive voltage divider and the operational amplifier (OA). The same current source was used to provide the reading and writing currents. The voltage difference of the $IrMn_3$ and Pt-only arms was used as the input to the OA. **b** Output voltage of the operational amplifier, showing an amplified switching signal of the device (red points correspond to a current pulse from the current source to ground, and blue points from ground to the current source).

We used this method in a Pt(5)/$IrMn_3$(10)/MgO device with an $IrMn_3$ pillar of 8 μm diameter. The resistances of the arms with and without the $IrMn_3$ pillar were 536 Ω and 505 Ω, respectively. The resistors R_1 and R_2 were 98 Ω and 97 Ω, respectively. Since the arms of the device were connected in parallel, the writing current was increased to ~ 40 mA, which corresponds to a current of approximately 20 mA in each arm (as in our original experiments). The OA used in this experiment had the option to apply an external nulling voltage to compensate for the internal offset between its input terminals. We used this external nulling voltage to compensate for the finite imbalance of the resistance ratios in the voltage divider circuit. The result is shown in Supplementary Fig. 9b, where the constant background of the output voltage has been subtracted to show the voltage variation at the OA output after the application of the write pulses.

The results in Supplementary Fig. 9b show a clear switching signal observed at the output terminal of the OA. The voltage swing at the output is approximately 100 mV, which is ~10^4× larger than the voltage swing obtained in the measurements without an amplifier in the main text (See Fig. 2a). Note, however, that the results shown in Supplementary Fig. 9b show the amplified difference of the two input voltages V_+ and V_-, hence the control voltage due to the Pt arm is intrinsically subtracted from that of the antiferromagnetic arm in this measurement.

Supplementary Note 8: Differential voltage measurement in a double-cross device with two nominally identical $IrMn_3$ pillars

In addition to the differential voltage measurements shown in the main text and the alternative differential voltage method explained in Supplementary Note 7, we also carried out switching experiments in a double-pillar device, as shown in Supplementary Fig. 10a, which had two nominally identical pillars of 4 μm diameter placed on both crosses. While this device does not allow for the built-in comparison to a Pt-only cross as in the previous case, it allows for a built-in subtraction of the readout signals from the two pillars. In this method, the same write current is applied through both pillars but with different directions, i.e. $I^{2\text{-}6}_{write}$ and $I^{5\text{-}3}_{write}$ or $I^{6\text{-}2}_{write}$ and $I^{3\text{-}5}_{write}$. This is followed by a measurement of the output voltage $V^{2\text{-}3}_{read}$ similar to the experiments described in the main text, which corresponds to the subtraction of the switching signals from the two pillars. The result is shown in Supplementary Fig. 10b, clearly indicating the expected switching behavior also in these differential measurements. Results plotted in Supplementary Fig. 10b are for a Pt(4)/$IrMn_3$(10)/MgO double-pillar device with a diameter of 4 μm (a microscope photograph is shown in Supplementary Figure 10a).

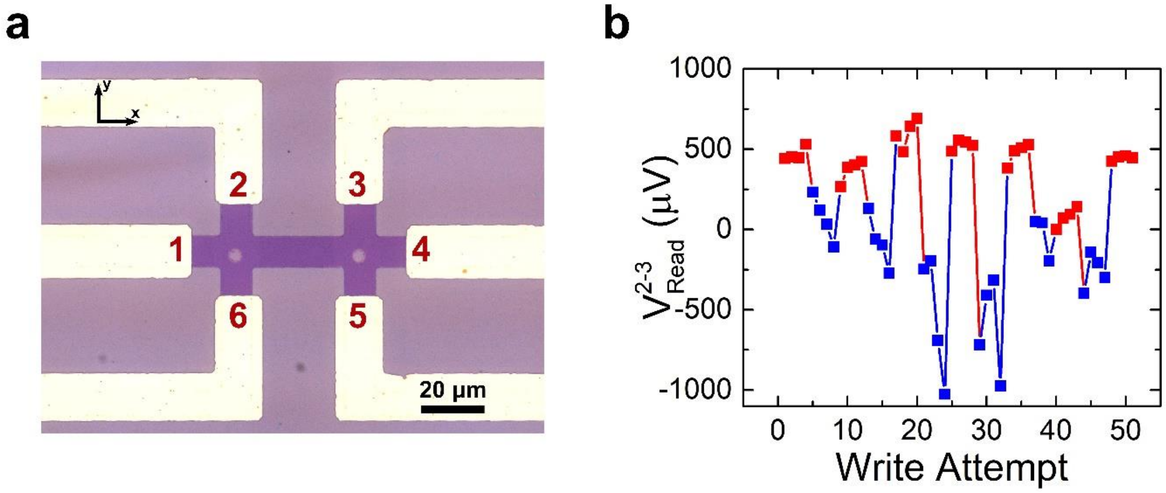

Supplementary Fig. 10. a Microscope photograph of the 4 μm diameter double pillar device measured using the double-pillar differential voltage measurement. **b** Readout voltage after two simultaneous writing currents of 20 mA amplitude are applied through both pillars at the same time but in opposite directions. Red points correspond to current directions $I^{2\text{-}6}_{write}$ and $I^{5\text{-}3}_{write}$ and blue points correspond to the readout voltage when the current pulses have directions $I^{6\text{-}2}_{write}$ and $I^{3\text{-}5}_{write}$.

Supplementary Note 9: Switching behavior after thermal annealing of the devices

The switching behavior of the $IrMn_3$ micropillars was studied after thermal annealing of the samples at 400 K under an applied out-of-plane magnetic field of 2 T. The sample was heated from room temperature (295 K) to 400 K at a ramp rate of 5 K/min and kept at 400 K for 5 min followed by cooling back to 295 K at a ramp rate of 5 K/min while a magnetic field of 2 T was applied throughout the duration of the temperature cycling. The thermal treatment was performed using a LakeShore CRX-VF probe station. The thermal treatment was carried out on the same double-pillar device shown in Supplementary Note 8, and the switching signal after the thermal treatment was measured using the double-pillar differential voltage method. The results are shown in Supplementary Fig. 11.

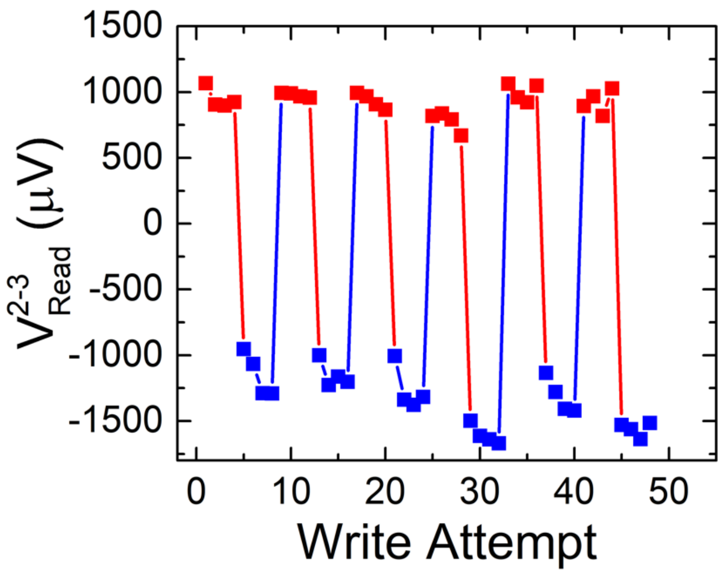

Supplementary Fig. 11. Measured voltage when two simultaneous pulses are applied to both pillars at the same time with directions I^{2-6}_{write} and I^{5-3}_{write} (red points), and I^{6-2}_{write} and I^{3-5}_{write} (blue points).

The measured output voltage after the thermal treatment shows an enhancement by ~2×. We ascribe this increase in the readout voltage to a possible increase in the domain size and reduction of domain wall pinning centers in our films subsequent to the thermal treatment, which results in a larger change of the average Néel vector in each pillar for the same amount of write current.

Supplementary Note 10: Two-sublattice model

The mesoscopic description of an antiferromagnet allows one to neglect the microscopic details of the sublattices and average them into only two magnetization sublattices which sum up to a null net magnetization. Therefore, the system can be described by means of two Landau-Lifshitz-Gilbert (LLG) equations [7, 8], strongly coupled through an exchange term,

$$\begin{cases} \dfrac{d\boldsymbol{m}_1}{dt} = -\gamma_0 \boldsymbol{m}_1 \times \boldsymbol{H}_{eff,1} + \alpha \boldsymbol{m}_1 \times \dfrac{d\boldsymbol{m}_1}{dt} + \boldsymbol{\tau}_{SH,1} \\ \dfrac{d\boldsymbol{m}_2}{dt} = -\gamma_0 \boldsymbol{m}_2 \times \boldsymbol{H}_{eff,2} + \alpha \boldsymbol{m}_2 \times \dfrac{d\boldsymbol{m}_2}{dt} + \boldsymbol{\tau}_{SH,2} \end{cases} \quad (1)$$

where γ_0 is the gyromagnetic ratio and α is the Gilbert damping parameter,

$$\boldsymbol{\tau}_{SH,i} = -\gamma_0 H_{SH}\, \boldsymbol{m}_i \times \left(\boldsymbol{m}_i \times \boldsymbol{p}\right) \quad (1)$$

is the Slonczewski-like SOT [9, 10] associated mainly with the spin-Hall effect [11, 12], with the amplitude given by $H_{SH} = \hbar \theta_{SH} J / \left(2et\mu_0 M_S\right)$. In the last expression, $\hbar$, θ_{SH}, $e < 0$, t, μ_0 are the reduced Planck's constant, the spin Hall angle, the electron charge, the AFM film thickness, and the vacuum permeability respectively, while J is the applied current density. The saturation magnetization is equal in both sublattices $M_{S1} = M_{S2} = M_S$. The direction of the spin polarization is $\mathbf{p} = \mathbf{z} \times \mathbf{j}$ (see Fig. 1 in the main text), $\boldsymbol{j}$ being the unit vector of the current density direction (*x* for horizontal pulses and *y* for vertical ones). Finally, $\mathbf{H}_{\mathrm{eff},1}$ and $\mathbf{H}_{\mathrm{eff},2}$ are the effective fields for the first and second sublattice, respectively. The main contributions to these fields are the exchange interaction and the cubic anisotropy. The former can be written as the sum of three terms

$$\boldsymbol{H}_i = \frac{2A_{11}}{\mu_0 M_S} \nabla^2 \boldsymbol{m}_i + \frac{4A_0}{a_m^2 \mu_0 M_S} \boldsymbol{m}_j + \frac{A_{12}}{\mu_0 M_S} \nabla^2 \boldsymbol{m}_j\,, \quad (1)$$

where $i \neq j$, a_m is the magnetic lattice constant and A_{11}, A_0, A_{12} are the inhomogeneous intra-lattice, homogeneous inter-lattice and inhomogeneous inter-lattice exchange constants characterizing the interaction. Finally, the cubic anisotropy field can be written as

$$\boldsymbol{H}_{anis} = m_u \left(m_v^2 + m_w^2\right) \boldsymbol{u} + m_v \left(m_u^2 + m_w^2\right) \boldsymbol{v} + m_w \left(m_u^2 + m_v^2\right) \boldsymbol{w}\ , \quad (1)$$

Where **u, v, w** are the unit vectors of the crystallographic reference system.

Supplementary Note 11: Micromagnetic description of pinning

For micromagnetic simulations, the pinning mechanism is assumed to originate from a granular microstructure where the grain size diameter is 15 nm, and each grain shows a different anisotropy constant. To mimic this mechanism, we generate a Voronoi tessellation with the corresponding grain size as shown in Supplementary Fig. 12a. The anisotropy constant of each grain is assumed to follow a Gaussian distribution with mean $K_c = -6.2 \times 10^6$ erg/cm^3 and standard deviation σ. Supplementary Fig. 12b depicts the probability distribution of a grain to have a certain anisotropy value for three different standard deviations. We consider different samples with standard deviation (normalized to the mean anisotropy constant) ranging from $\sigma = 0.02$ to $\sigma = 0.15$. Supplementary Fig. 12c shows the numerical computed threshold currents as a function of the standard deviation. Particularly, a standard deviation of the anisotropy constant of ~0.02 is needed to match the experimental threshold current density value of ~36 MA/cm^2. In the case of the pillar shown in the main text, we have considered the same parameters for the grain size and anisotropy mean value, while we chose the standard deviation to be $\sigma = 0.15$ in order to speed up the ground state study.

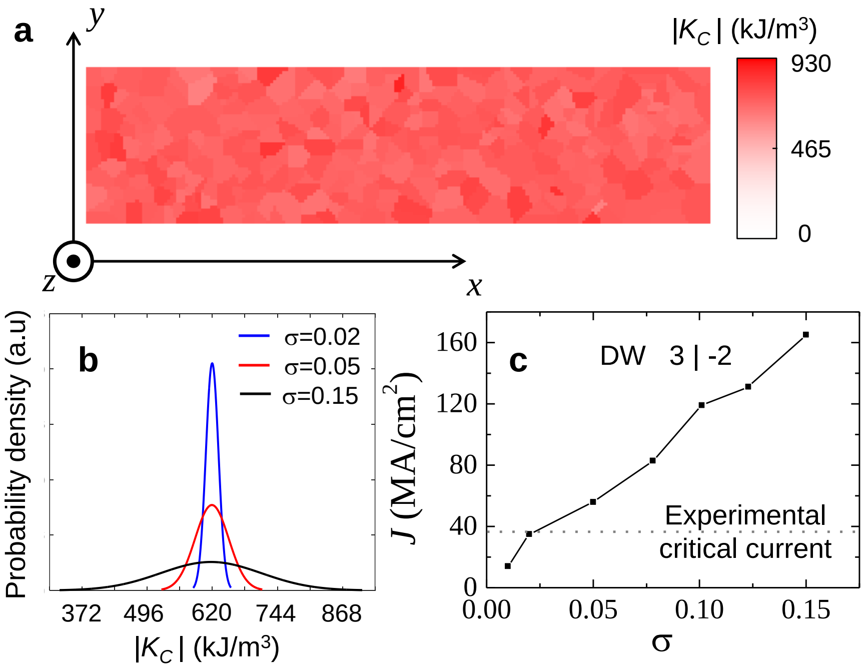

Supplementary Fig. 12. Pinning effects of a Voronoi tesselation of grains with different anisotropy values. **a** Voronoi tesselation of an $IrMn_3$ stripe. **b** Probability density of a grain to have a certain anisotropy value for three different standard deviations. **c** Threshold current of a 3|-2 DW as a function of the standard deviation. Dotted line represents the experimental threshold current density $J \approx 36\,\mathrm{MA/cm^2}$.

References

1. Zhou, J., et al., *Magnetic asymmetry induced anomalous spin-orbit torque in IrMn.* Physical Review B, 2020. **101**(18): p. 184403.
2. Kohn, A., et al., *The antiferromagnetic structures of IrMn3 and their influence on exchange-bias.* Scientific Reports, 2013. **3**(1): p. 2412.
3. van Dijken, S., J. Moritz, and J.M.D. Coey, *Correlation between perpendicular exchange bias and magnetic anisotropy in IrMn/[Co/Pt]$_n$ and [Pt/Co]$_n$/IrMn multilayers.* Journal of Applied Physics, 2005. **97**(6): p. 063907.
4. Baltz, V. and B. Dieny, *Influence of Pt as Mn diffusion barrier on the distribution of blocking temperature in Co/(Pt)/IrMn exchange biased layers.* Journal of Applied Physics, 2011. **109**(6): p. 066102.
5. Sort, J., et al., *Enhancement of exchange bias through a non-magnetic spacer.* Journal of Magnetism and Magnetic Materials, 2004. **272-276**: p. 355-356.
6. Sort, J., et al., *Tailoring perpendicular exchange bias in [Pt/Co]-IrMn multilayers.* Physical Review B, 2005. **71**(5): p. 054411.
7. Landau, L.D. and E.M. Lifshitz, *On the theory of the dispersion of magnetic permeability in ferromagnetic bodies.* Physik. Zeits. Sowjetunion, 1935. **8**: p. 153.
8. Gilbert, T.L., *A phenomenological theory of damping in ferromagnetic materials.* IEEE Transactions on Magnetics, 2004. **40**(6): p. 3443-3449.
9. Slonczewski, J.C., *Current-driven excitation of magnetic multilayers.* Journal of Magnetism and Magnetic Materials, 1996. **159**(1): p. L1-L7.
10. Berger, L., *Emission of spin waves by a magnetic multilayer traversed by a current.* Physical Review B, 1996. **54**(13): p. 9353-9358.
11. Dyakonov, M.I. and V.I. Perel, *Current-induced spin orientation of electrons in semiconductors.* Physics Letters A, 1971. **35**(6): p. 459-460.
12. Hirsch, J.E., *Spin Hall Effect.* Physical Review Letters, 1999. **83**(9): p. 1834-1837.
